# Combining old and new concepts in targeting telomerase for cancer therapy: transient, immediate, complete and combinatory attack (TICCA)

**DOI:** 10.1186/s12935-023-03041-2

**Published:** 2023-09-07

**Authors:** Jaber Haj Ali, Michael Walter

**Affiliations:** 1 Institute of Laboratory Medicine, Clinical Chemistry and Pathobiochemistry, Charité Universitätsmedizin Berlin, Augustenburger Platz 1, 13353 Berlin, Germany; 2 Institute of Clinical Chemistry and Laboratory Medicine, Universitätsmedizin Rostock, Ernst-Heydemann-Straße 6, 18057 Rostock, Germany

**Keywords:** Telomerase, Cancer therapy, Cancer, TICCA, Telomere, Transient immediate complete and combinatory attack

## Abstract

Telomerase can overcome replicative senescence by elongation of telomeres but is also a specific element in most cancer cells. It is expressed more vastly than any other tumor marker. Telomerase as a tumor target inducing replicative immortality can be overcome by only one other mechanism: alternative lengthening of telomeres (ALT). This limits the probability to develop resistance to treatments. Moreover, telomerase inhibition offers some degree of specificity with a low risk of toxicity in normal cells. Nevertheless, only one telomerase antagonist reached late preclinical studies. The underlying causes, the pitfalls of telomerase-based therapies, and future chances based on recent technical advancements are summarized in this review. Based on new findings and approaches, we propose a concept how long-term survival in telomerase-based cancer therapies can be significantly improved: the TICCA (Transient Immediate Complete and Combinatory Attack) strategy.

## Historical background

In 1994, telomerase activity has been identified in approximately 90% of human malignant tumors [1]. This finding had a long history of discoveries. In 1911, Alex Carrel, the winner of the Nobel Prize in physiology in 1912, hypothesized the existence of cellular immortality in culture [2]. He suggested that heart tissue cells can be maintained in long-term cultures by the simple renewal of the culture medium. This concept turned out to be wrong later as the used cell culture medium in these experiments continuously reseeded fetal cells. It initiated, however, numerous further experiments in this field. In 1938, Herman J Muller noticed X-ray-resistant cap-like structures at the chromosomal ends that he called “telomeres” [3]. Barbara McClintock described chromosome end fusions and explained the crucial role of telomeres for chromosomal integrity [4]. Leonard Hayflick demonstrated in 1961 for the first time that human fetal cells in culture possess a limited ability to divide approximately 40 to 60 times, before they enter a state called replicative senescence [5]. The underlying end-replication problem was described in 1971 by James Watson [6]. Watson also speculated about a maintenance mechanism that may conserve the chromosomal ends. Alexsey Olovnikov postulated that successive telomere attrition may result in gene damage, contributing to cellular and human aging [7]. Tetrahymena thermophila telomere tandem repeats have been sequenced in 1978 by Elizabeth Blackburn and Joseph Gall. They found out that Tetrahymena telomeres contain 20–70 hexanucleotide copies with the sequence 5′-CCCCAA-3′ on one strand and 5′-TTGGGG-3′ on the complementary strand [8]. The discovery of telomerase in 1985 by Elizabeth Blackburn and Carol Greider was the scientific basis for the Nobel Prize in 2009 together with Jack W. Szostak. During the next years, it was shown in many studies that chromosomal integrity is protected by telomerase [9]_._ Human telomerase was further characterized by Gregg Morin. He described in HeLa cells the repetitive TTAGGG motif specific for human telomeres [10]. In 1998, the Shay and Wright group was able to immortalize human cells by introducing the catalytic subunit of human telomerase (hTERT) into normal human cells [11].

## Physiology and pathophysiology

### Telomeres and the telomere complex

**Fig. 1:**
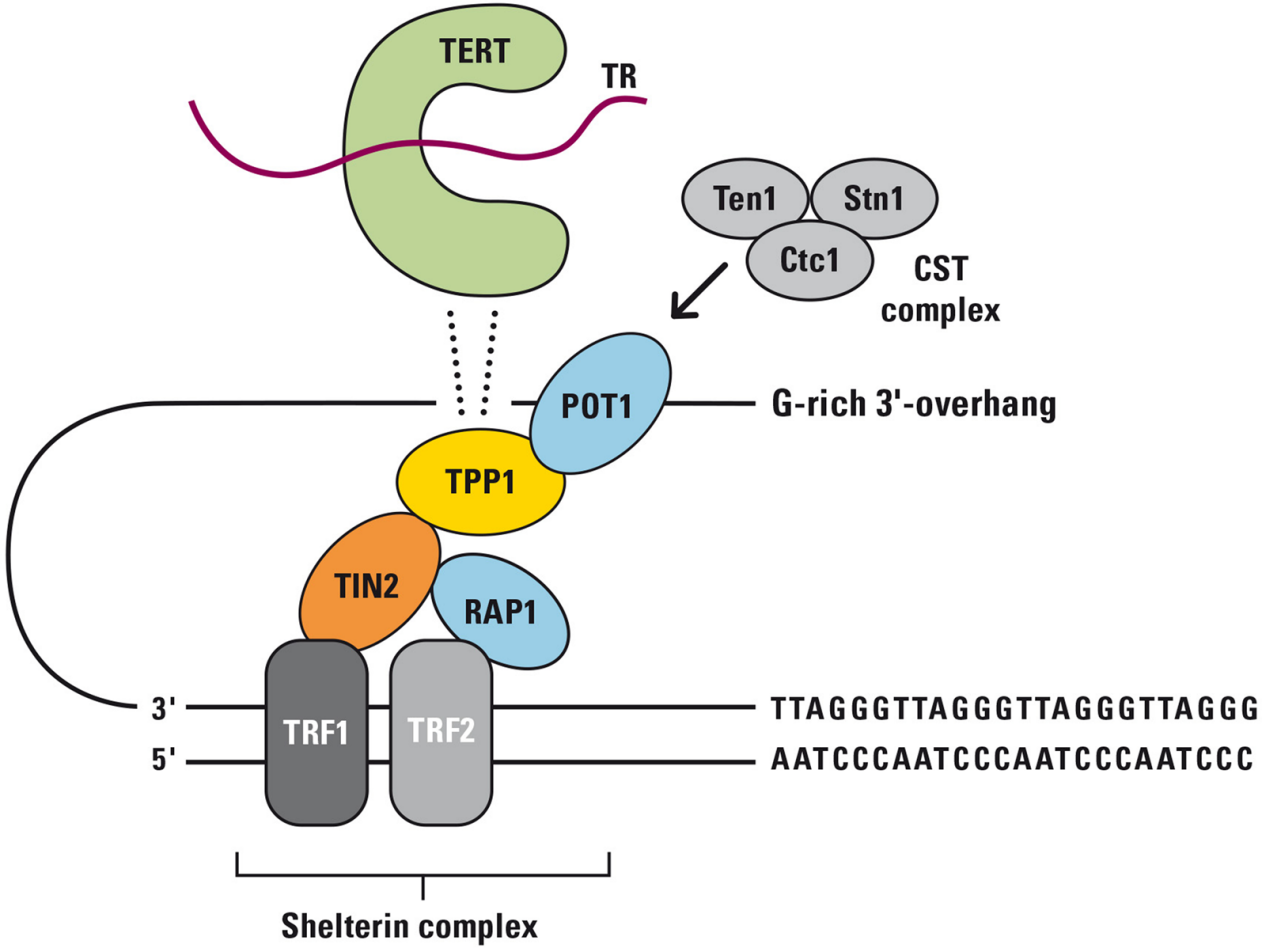
Schematic description of the shelterin complex. Telomeres end with a G-strand 3’ overhang that invades the double-stranded telomeric sequence. A closed structure called telomere loop (T-loop) is formed. The shelterin complex coordinates the T-loop formation and protects the end of the chromosome from damage. Telomeric Repeat Factor 1 and 2 (TRF1 and TRF2) bind to double strand (ds) DNA and form two separate complexes with other proteins. Protection of telomeres protein 1 **(**POT1) recognizes single-stranded DNA while Repressor/activator protein 1 (RAP1) binds to DNA at the ds-ss joint. TRF1- and TRF2-Interacting Nuclear Protein 2 (TIN2) binds TRF1 and TRF2 spontaneously and protects TRF1 from being degraded. TINT1/PTOP/PIP1 protein (TPP1) and POT1 form a heterodimer. TPP1 also links TIN2 and POT1 and recruits telomerase (TERT) with its telomerase RNA component (TR) to the shelterin complex. TPP1 contains two telomerase binding regions. The CST complex has three components: conserved telomere protection component 1 (CTC1), suppressor of cdc thirteen 1 (STN1) and telomeric pathway with STN1 (TEN1), which are thought to function in telomere lagging-strand synthesis. The CST complex binds to newly synthesized repeats and blocks telomerase activity

Telomeric stability at the ends of eukaryotic chromosomes is formed via a structure composed of telomeric hexanucleotides (TTAGGGs in human beings, forming a total of up to 15 kb in germ line cells) and a protein complex called shelterin complex (Fig. 1). This telomere structure is highly regulated by several regulatory mechanisms and pathways [11]. According to current concepts, replicative aging (triggered by telomere shortening) is a species-specific mechanism with a tumor-suppressor function in many large long-lived organisms [12]. Regular senescence limits the maximum number of cell divisions and may thus limit the probability of tumorigenesis and inhibit the replication of abnormal chromosomes. There is also some evidence that telomere shortening also activates protective genes that have a tumor-suppressive effect [13] and that this process is disturbed in genetic instability syndromes [14]. On the other hand, excessive and uncontrolled telomere attrition and telomere damage may also make cells more vulnerable to genomic instability, which then may result in bypassing of the p53/p21/p16/pRb tumor suppressor pathways. Consequently, malignancy can result from the incorrect removal of senescent cells.

Due to the failure of DNA polymerase to replicate the very telomere ends (the so-called end replication problem), telomeres become shorter with each cell division [15]. Telomerase is a specific reverse transcriptase enzyme that can overcome the end replication problem by use of a unique structure consisting of two subunits: TR (telomerase RNA component), which serves as an template for telomere replication, and hTERT (human telomerase reverse transcriptase). hTERT acts as the catalytic subunit and elongates chromosome telomeres on the RNA template TERC [16]. A substantial activity of telomerase is restricted to cells needing high replicative capacity such as stem cells and progenitor cells.

Telomeres are a target for both anti‐cancer and cell rejuvenation applications. Telomere shortening can have both positive aspects, namely tumor-suppressor effects by inducing replicative senescence; and negative aspects for chronic diseases and even cancer at extreme telomere attrition. Extreme telomere attrition may lead to genetic instability and chromosome fusions with negative effects for almost all chronic diseases including cancerogenesis. Inflammatory processes and other forms of stress can accelerate telomere attrition in immune cells by increasing leukocyte turnover and replicative exhaustion associated with impaired immune functions. Moreover, higher levels of cytokines may directly attack telomere structures and may also affect telomerase activity and thus telomere length [17].

### Oxidative stress and inflammation synergistically promote telomere loss

Oxidative damage at telomeres and the chronic activation of inflammatory responses lead to accelerated telomere attrition and potential implications for cellular aging, disease development, and overall health [18, 19]. Oxidative stress occurs in situations of imbalances between the production and neutralization of reactive oxygen species (ROS) and the ability of cells to repair the resulting damage. ROS can be generated during normal cellular metabolism and in response to external stressors such as environmental toxins, radiation, or inflammation. ROS directly damages telomeric DNA by inducing oxidative modifications, including DNA strand breaks and base modifications. Telomeres are particularly susceptible to oxidative damage due to their guanine-rich repetitive sequences, which are more prone to oxidation.

Inflammation is a complex biological response of the immune system to combat infection, injury, or disease. Chronic or persistent low-grade or systemic inflammation, can have detrimental effects on cellular health and may contribute to telomere loss in human beings [20]. Inflammatory processes involve the release of pro-inflammatory cytokines and the activation of immune cells. These immune responses can generate ROS as byproducts, further exacerbating oxidative stress and telomere attrition. Activation and replication of lymphocytes leads to a senescence phenotype termed as senescence-associated secretory phenotype (SASP) [21] and ultimately to an exhaustion of stem cell pools, which further contributes to immune cell senescence and compromised immune function. Altogether this overload of oxidative stress results in telomere shortening despite the fact that inflammatory mediators and cytokines initially even trigger telomere elongation by activating telomerase. Moreover inflammatory signals such as nuclear factor-kappa B (NF-κB) can activate genes involved in short-term cell proliferation and survival but rather suppress genes associated with telomere maintenance and DNA repair. This hyperproliferative imbalance contributes to telomere attrition, genomic instability and a process called geroconversion [22, 23].

### Induction of telomerase in cancer cells

Telomerase in cancer cells is regulated at genetic, epigenetic, transcriptional, post-transcriptional and post-translational level [24]. In normal human cells, telomerase activity appears is strictly regulated during development. Telomerase activity is down regulated during early embryogenesis and cell differentiation in most somatic cells but remains active in male germ cells, activated lymphocytes, and stem cell populations [25]. These observation show that tissues with high proliferative potential need telomerase to maintain telomere length and genetic stability. In tumor cells, however, hTR and hTERT containing chromosomal regions can be amplified [26–31], or chromosomal rearrangements may direct the promoter away from a stringent repressive epigenetic environment or to the proximity of enhancers [32]. The hTERT subunit of telomerase is upregulated in most cancer cells including lung tumors, neuroblastoma, breast carcinomas, cervical carcinomas, and hepatocellular carcinoma. Telomerase can also play a regulatory role in the metastasis of cancer cells [33]. Epigenetic H3-K9Me marks in hTR or hTERT result in lower gene expression levels while H3-K4Ac, H4-K4Ac, H3-K9Ac and H3-K4Me marks are associated with higher hTERT expression [34]. A wide variety of genetic polymorphisms of telomerase complex constituents results in elongated telomeres and a higher cancer risk [35–38]. Transcription factors modulate the telomerase activity in cancer cells: The hTR gene promoter is activated by NF-Y, Sp1, pRB and HIF1; it is suppressed by Sp3 and signaling pathways such as JNK that cause a switch from Sp1 to Sp3 promoter binding [39–41]. Promoter binding sites for c-Myc, HIF1, ETS, E2F and Sp1/Sp3 integrate hTERT transcriptional responses with dysregulated pathways in tumor cells [42–44]. Telomere shortening is associated with hTERT mRNA induction [45] and modulation of key growth signaling pathways [13]. Most oncogenic growth promoting pathways activate telomerase expression, whereas pathways controlling growth suppression, cell death and senescence have the opposite effect. For example, MAPK increases hTERT expression [46] and certain hTERT promoter mutations generate binding sites for ETS family transcription factors [47, 48].

Other hTERT activation pathways in cancer include CDK2, CDK4 [49] and AKT [50] dependent signaling and deregulated repression pathways dependent on TGFβ [50], TNFα [51], and other cell cycle inhibitors [52]. Disrupted developmental pathways such as Wnt may induce hTERT expression [52–55], and multiple kinases participate in pathways upstream of hTERT regulation [41, 56].

### Induction of alternative lengthening of telomeres (ALT) in cancer cells

A subset of dividing cells can maintain telomere length in the absence of telomerase. This observation led to the discovery of a telomerase-independent telomere maintenance mechanism called ALT (Alternative Lengthening of Telomeres), which arises in cells at a state called crisis [57]. Genomic instability is a feature of all ALT-positive tumors. The inhibition of telomerase provokes the ALT phenotype in tumor cells as an alternative means of telomere maintenance. Most ALT + cancers are of mesenchymal or neuroendocrine origin and (except HER2 + breast carcinoma) not of epithelial origin. The prevalence of ALT positivity is dependent on the tissue and greatly varies from 1% in bladder cancer to 30% in glioma and 65% in anaplastic astrocytoma [58]. In a study comprising 6110 primary tumors from 94 different cancer subtypes ALT was observed in 3.7% of all tumor specimens, but was not observed in benign neoplasms or normal tissues [59]. The frequency of ALT is estimated to occur in about 10–20% of all tumors in other studies [58, 60]. The varying prevalences are due to the fact that confirming neoplasms as ALT + is not as simple as defining their TEL + counterparts. There is no apparent singular enzyme or characteristic to rely on. Thus identification of ALT depends on a tumor displaying multiple of ALT’s characteristics such as (i) absence of telomerase activity, (ii) presence of telomeres of substantial (> 50 kb) and heterogeneous length (< 8 kb and > 50 kb), (iii) elevated levels of telomere sister chromatid exchange, (iv) extra-chromosomal telomeric repeats or C-circles, (v) telomere dysfunction-induced foci (TIFs) [58, 61]. ALT was found to be associated with an increased risk of death in patients with sarcoma [62], in HER2 + breast carcinoma, in angiosarcoma, pancreatic neuroendocrine tumors, liposarcoma and leiomyosarcoma but not in glioma, neuroblastoma and osteosarcoma, in which cancer types ALT + tumors had a better prognosis than TEL + tumors [58]. These observations highlight the diverse nature of telomere maintenance mechanisms and their activation in different tissues, underscoring the need for further research to elucidate the underlying factors contributing to such tissue-specific variations.

The mechanisms by which telomerase inhibition promotes the ALT phenotype are not fully understood. The inhibition of telomerase activity can create a cellular environment conducive to ALT activation. The altered dynamics of telomere lengthening and shortening in telomerase-negative immortal cancer cells suggest that ALT maintains telomere length using a DNA recombination-mediated mechanism. The repetitive telomeric repeats may act as copy templates, with one telomere molecule acting as a donor and another the recipient. This may occur by strand invasion and a copy process between sister telomeres or between telomeres on different chromosomes. Additionally a telomere may loop back and invade its own telomeric region (forming a T-loop) providing its own copy template or extrachromosomal telomeric DNA may provide the required template ALT may trigger a feedforward loop to recruit Bloom syndrome-associated helicase BLM, which participates in telomere replication [63].

Not all cancer cells are capable of activating ALT in response to telomerase inhibition. The propensity to undergo ALT may vary depending on the specific genetic alterations and molecular characteristics of the cancer cells. Aberrations in the ATRX-DAXX-H3.3 pathway correlate with the ALT positivity. Loss of ATRX and DAXX increases the likelihood for ALT positivity to 83% in leiomyosarcoma and to 100% in osteosarcoma and liposarcoma.

### Alternative splicing of human telomerase

The human hTERT gene (42 kb) is located on chromosome 5p15.33, spans 16 exons and 15 introns, and encodes (from the full length transcript) an active 1132-amino acid (127-kDa) protein. The expression and activity of hTERT are regulated at various levels including promoter organization, mRNA splicing, post-translational modification, protein folding, and interaction with other proteins [64]. The regulation by alternative splicing of pre-mRNAs is critical because small amounts of TERT may have significant cellular consequences. [65]. Telomerase activity is strongly dependent on the relative expression level of the full-length hTERT variant, which varies from 1 to 90% [65–68]. The modulation of telomerase activity through alternative splicing is involved in embryonal development, cell differentiation and cancerogenesis [69] but its exact physiological function is poorly understood. At least 22 different splice variants of hTERT mRNA have been identified [70, 71]. The regulation of alternative splicing can occur through specific splicing factors. These factors can bind to regulatory elements within the TERT gene or its pre-mRNA and influence the inclusion or exclusion of exons during splicing. Cellular signaling pathways and environmental cues can also modulate the activity or expression of splicing factors, thereby indirectly modulating telomerase activity. One of the key alternative splicing events in TERT mRNA involves the inclusion or exclusion of exon α. The presence or absence of this exon leads to the generation of two major isoforms known as α + and α-. The α + isoform is the full-length form of hTERT, capable of elongating telomeres effectively. The α- isoform lacks a segment necessary for its catalytic activity and acts as a dominant-negative protein that can bind to hTR but cannot maintain telomeres [72]. Consequently, cells expressing the α- isoform exhibit reduced telomerase activity and shorter telomeres. This truncated variant is abundant in cancer cells [73] and in activated lymphocytes [74]. Combinations of other splice variants (α + β − and α − β +) have been described in different cell types with a 1–15% range [73].

### Interrelationships among mitochondrial and telomere function and involvement of alternative splicing

The function of the many hTERT mRNA splice products is not known. In this context it is of interest that an apoptotic endonuclease G (EndoG) upregulates the inactive alternatively spliced telomerase variant and thus indirectly inhibits its enzymatic activity in CD4 + human T lymphocytes, associated with induction of replicative senescence [74]. In general, resistance to apoptosis in human cells is conferred by adequate telomerase function and telomere stability [75]. On the other hand, an inactive telomerase splice variant (which is associated with lower telomerase activities) can also protect cells from apoptosis [76], pointing to a complex link between mitochondrial and telomere function. It is important to know such connections, since therapeutical induction of β- variant (to inhibit telomerase) may face an opposite effect when cancer cells become more resistant to apoptosis induction [76] or less sensitive to irradiation [77]. Moreover the N-terminal region in hTERT contains a BH3-like motif found in antiapoptotic BCL-2 family proteins, further suggesting a close functional link between hTERT and the mitochondrial pathway of apoptosis [78]. Apparently there is a close interrelationship among mitochondrial metabolic stress, associated with reactive oxygen species (ROS) production, and replicative senescence, triggered by telomere shortening. This is plausible insofar as both telomeric and mitochondrial DNA damage can result in senescence and apoptosis and both types of DNA share common defence strategies such as G-quadruplexes, D-loops, RNA:DNA heteroduplexes, epigenetic marks, or supercoiling. In many situations oxidative stress induces dual injury via crosstalk between telomeres and mitochondria [79]. To avoid replication stress both compartments use similar enzymatic strategies including endonucleases, topoisomerases, helicases, or primase, and key telomeric proteins, such as hTERT, hTR/hTERC (human telomerase RNA component) and the sheltering complex protein TIN2 shuttle from telomeres to mitochondria and may modulate mitochondrial metabolism and the production of ROS in a reciprocal feedback manner.

Altogether these findings suggest that the fine tuning of whether a cell undergoes senescence, apoptosis or regeneration is closely coordinated between the mitochondrion and the telomere. Alternative splicing of hTERT may play an important regulatory role in this process. Better knowledge of this regulation is imortant to enable the development of more specific telomerase inhibitors.

## Utility in cancer therapy

Telomerase activity is high and its components are up regulated in cancer cells [1]. Fortunately, inhibition of telomerase expression or activity in tumor cells does not significantly influence healthy cells [80]. Thus telomerase is a potent target to treat malignancies [81]. Telomerase can also be used for rejuvenation procedures to counteract telomere attrition in pre-senescent cells [81, 82]. Potential mechanisms of antitumor and rejuvenation therapies include inhibition/activation of gene transcription, inhibition/activation of protein synthesis, modulation of activity by posttranslational modifications, modulation of telomerase activity by cellular sequestration, interference with telomerase complex assembly, modulation of signaling pathways and molecules involved in enzyme activation, and modulation of telomerase complex catabolism including vaccine therapy [81]. From a technical point of view, there are several strategies for the telomerase-based treatment of cancer: oligonucleotide inhibitors, small-molecule telomerase inhibitors, immunotherapeutic approaches, telomerase-directed gene therapy, phytochemicals and various other substances with off-target effects and a broad range of mechanisms.

### Oligonucleotide inhibitors

**Fig. 2:**
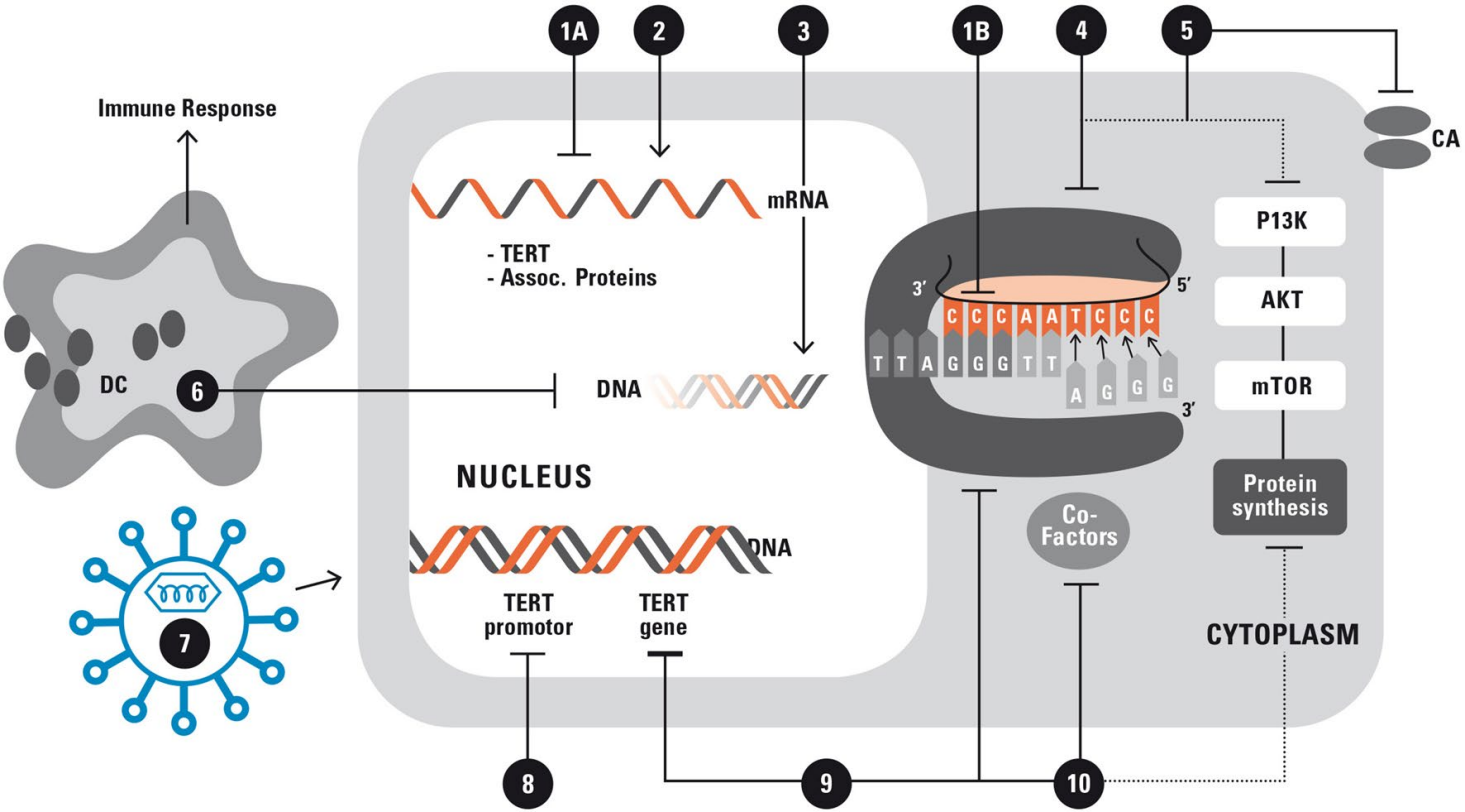
Strategies for telomerase-based treatment of cancer. The figure summarizes the traditional and novel strategies for telomerase-based treatment of cancer: (1) oligonucleotide inhibitors inhibiting telomerase by binding to the mRNA of telomerase components and to turn the gene "off" (1A) or by inactivating the RNA template of the telomerase complex (1B*)*, (2) alternative splicing to induce splicing patterns with inactive variants, (3) quadruplex stabilizers to inhibit telomerase indirectly, (4) small-molecule telomerase inhibitors with a broad variety of targets, (5) dual hybrid telomerase inhibitors with different tumor suppressive effects in one hybrid molecule such as telomerase inhibition plus carboanhydrase (CA) inhibition, (6) immunotherapeutic approaches as* v*accine synthesis against telomerase or as adoptive cell therapy by modification of lymphocytes ex vivo, (7) viral vector mediated delivery (of Cas9-sgRNA) for mutational repair (AAV) or oncolytic therapy (HSV) in ALT positive tumors, (8) telomerase-directed gene therapy by directly addressing the telomerase gene and promotor and selective induction of high concentrations of cytotoxic and oncolytic proteins*,* (9) phytochemicals and various other substances with a broad range of mechanisms, and (10) direct attack of the shelterin complex. These strategies might be combined with novel technologies such telomere deprotection, Crisp/Cas9 induced abrupt telomere attrition and use of transiently effective vector systems to avoid negative effects of the conventional methods such as long start-up time and negative side-effects on the immune system with long-term treatment

In cell culture models, antisense oligonucleotides and chemically-modified nucleic acids were shown to inhibit telomerase by binding to the mRNA of telomerase components (hTERT and associated proteins) and to turn the gene “off” (1A in Fig. 1) or to inactivate the RNA template of the telomerase complex [83] (1B in Fig. 1) both resulting in telomere attrition and ultimately senescence and/or apoptosis. The 13-mer N3’-N5’ thio-phosphoramidate oligonucleotide oligonucleotide inhibitor imetelstat (by Geron Corporation, Menlo Park, CA, USA) binds to the template region of TERC [84], resulting in direct, competitive inhibition of telomerase enzymatic activity, rather than inhibition of protein translation. Its nucleic acid backbone provides resistance to cellular nucleases, and improves binding affinity to the target, whereas a lipid group enhances cell permeability. Imetelstat (GRN163L) was successful in the treatment of glioblastoma tumors [85]. In principle, treatment is always possible when a cancer type permits sufficient time to grow for the erosion of telomeres to critical levels to trigger cellular senescence. Clinical studies for breast and lung cancers are underway. Severe side effects were not observed in these studies so far and possible combinatory therapies with established therapies are in preparation (1 in Fig. 2).

### Small-molecule telomerase inhibitors

A small molecule is a low molecular weight organic compound (molecular weight < 1000 Dalton) involved in biological processes. Small-molecule telomerase inhibitors have been identified by use of chemical libraries or were synthesized based on the structure of natural telomerase inhibitors such as epigallocatechin-3-gallate (EGCG) [86]. Some targets of small molecules have overlapping functions [87].

Several small molecule chemical compounds were shown to modulate aberrant splicing [88]. Both natural products derived primarily from bacteria and chemical synthesis provided leads for therapeutic compounds targeting splicing [89]. Other small molecule inhibitors are G4-stabilizing compounds or are noncompetitive inhibitors or inhibitors with a wide variety of different, in part off-target, effects such as many phytochemicals (4 in Fig. 2).

**BIBR1532:** The synthetic compound BIBR1532 is a selective noncompetitive small molecule hTERT inhibitor [90, 91]. It binds to the active site of telomerase and interferes with its catalytic activity [92]. It inhibits telomerase in leukemia cell lines [92–95] and had promising anticancer effects in first preclinical studies [96, 97]. Moreover it can induce cell sensitivity to chemotherapy in MCF-7 and breast cancer stem cells [97]. Treatment leads to telomere attrition and inhibition of cell proliferation in gliobastoma cells [98] without obvious unspecific effects on DNA and RNA polymerase. It has been shown that this approach is able to induce apoptosis and to modulate mTOR signaling [97]. BIBR1532 has become a lead component to design new selective telomerase inhibitors. On the other hand, there are significant concerns regarding its toxicity [99], and this agent has not yet been evaluated in larger clinical trials.

**G Quadruplex stabilizer:** G4-stabilizing compounds are an own class of small molecules that stabilize G-quadruplex (G4) structures, e.g. secondary DNA or RNA structures formed by guanine-rich sequences. G4 structures are enriched in telomeric DNA but also elsewehere in promotor regions where they alter gene expression at many different levels and act as a key regulatory element perturbing the nature of epigenetic marks and chromatin architecture [100]. The single stranded telomeric DNA folds into G-quadruplex structures and this three-dimensional structure prevents telomerase-telomere interaction and indirectly inhibits telomerase activity. G-quadruplex stabilizers are therefore potential anti-cancer therapeutics.

A wide variety of G4 stabilizers have been described including natural products and the corresponding synthetic analogs. Most G4 stabilizers are flat aromatic molecules and their modes of action are through π–π and electrostatic interactions. Bisamidoanthraquinone was the first G4 ligand [101], which was further modified to BSU6039 and Braco-19 with reasonable telomerase inhibitor activities in the 100 nM IC50 range. BRACO-19 has been studied for its potential as an anticancer agent by blocking telomerase activity and inducing telomere dysfunction [102, 103]. The spectrum also encompasses multissubstituted derivatives, perylene derivatives and naphthalene diimides such as PIPER and Tel10 with upto 50-fold binding preference for G4 [104]. Pyridostatin (PR82) is a synthetic compound that stabilizes G4 structures and induces severe telomere dysfunction (Müller et al., 2012). Various preclinical models have proven anticancer effects. For example, pyridostatin is a strong candidate drug for targeting BRCA1/2‐deficient tumours [105].

Telomestatin is a macrocyclic compound that was first isolated from the bacteria *Streptomyces anulatus* [106]. Telomestatin induces the formation of basket-type G-quadruplex (G4) structures in the telomeric region, associated with a decrease in the activity of the telomerase and premature senescence [107]. It was shown to disrupt telomeric structures inglioma cells [108].

RHPS4 (also known as telomestatin derivative) is a synthetic derivative of telomestatin with improved solubility and stability while maintaining high affinity for G4 structures. Treatment of uterus carcinoma cells cells with RHPS4 leds to the displacement of the telomerase catalytic subunit (hTERT) from the nucleus and induction of telomere-initiated DNA-damage signalling. A combination of RHPS4 with the mitotic spindle poison Taxol caused tumour remissions and further enhancement of telomere dysfunction [109].

A more recent trend is the development of compounds by chemical splicing. Metal–organic complexes as G4 binders have improved biological activity [109–117]. In these substances a metallic center is located on a cationic quadruple chain, which forms an optimum structure for substance-G4 interactions. The first metal containing ligand was a porphyrin based compound. The introduction of a metallic element (Cu, Ni, Mn) may reduce telomerase inhibitory effect but improves the selectivity (preference for G4) [118]. Transition metal complexes are to date the strongest ligands with upto 10 000-fold preference for G4 with lower or medium level telomerase inhibition activity. Apparently the geometry of the metallic center is a key parameter in controlling selectivity [118].

Despite these first successes most G quadruplex stabilizer have problems in either the strength of stabilization or the selectivity (3 in Fig. 2).

### Immunotherapeutic approaches

Vaccine synthesis against the active site of telomerase is a potential immunotherapeutic approach [119]. The use of adoptive cell therapy, e.g. the extraction of T lymphocytes cells from the patient, their modification in vitro and return to the same patient, has successfully been used in prostate adenocarcinoma in mice [120]. hTERT immunotherapy has been used as anticancer strategy in melanoma, acute myeloid leukemia, glioblastoma, prostate, renal, pancreatic, hepatocellular, and non-small-cell lung cancer in 18 phase I-III studies [121] Moderately improved survival rates have been observed in pancreatic cancer patients (with the substance GV1001) [122] and in hepatocellular carcinoma patients [123]. Median survival ranged from 88 to 450 days in non-responders and from 216 to > 600 days in responders [122–129].

Limited success in studies could be due to the induction of self-tolerance, limited T-cell repertoire, negative effects of the immunosuppressive tumor microenvironment, and unknown individual dissimilarities. Some of the proposed future studies designed for improvements focus on; (a) the enhancement of synergy between CD8^+^ and CD4^+^ cells via immunization with both, type I and II MHC hTERT peptides, to improve the number of memory CD8 + cells; (b) the decrease of immune-tolerance by immunization with low affinity (mutant) MHC I hTERT peptides and additional immunization with peptides of non-self-antigens and (c) escaping the development of undesired effects of immune-suppressive cancer microenvironments by personalized approaches with a focus on patients with early stage cancer [121] (6 in Fig. 2).

### Telomerase-directed gene therapy

In telomerase-directed gene therapy trials, the telomerase promoters of cancer cells are potential targets. In this approach, cancer cells with a high telomerase expression are selectively killed by directly addressing the telomerase gene and promotor and selective induction of high concentrations of cytotoxic and oncolytic proteins [130] without harming normal cells (8 in Fig. 2).

### Alternative splicing

Several small molecule chemical compounds were shown to modulate aberrant splicing [88]. Both natural products derived primarily from bacteria and chemical synthesis provided leads for therapeutic compounds targeting splicing [89]. For example, 12459, a G-quadruplex-interacting agent suppresses full-length hTERT and telomerase activity and induces inactive β- variant in the A549 lung carcinoma cell line [131]. Apparently the substance binds to G-rich sequences that can form G-quadruplexes thereby affecting alternative splicing toward β- formation. A similar approach was performed with compound CX-5461, which stabilizes the G-quadruplex [132] and shifts hTERT splicing pattern to an increase in β- and a decrease in the full-length expression, associated with telomerase inhibition and induction of apoptosis in glioblastoma cell lines.

The low selectivity of compounds targeting the spliceosome is problematic in modulating hTERT alternative splicing. To overcome the low selectivity in targeting the spliceosome compounds were developed that selectively switch the full-length active hTERT variants to any inactive variant. Such splice-switching oligonucleotides (SSOs) are short, modified antisense nucleic acids that block the RNA–RNA base-pairing or protein–RNA binding interactions of the splicing machinery [133]. The possible usability as therapeutics was already shown by successful splice switching in the DU145 prostate cancer cell [134]. A switch from the full-length hTERT toward the α-β- variant led to the reduction of telomerase activity and apoptosis, possibly complementary or predominantly caused by disruption of regular telomere cap function [135]. Moreover the blocking of cis-regulating elements within a binding site for splicing-regulatory proteins was addressed [135, 136] in more recent experimental settings. In a nonsmall cell cancer line modulation of a binding site for NOVA1 splicing-regulatory proteins resulted in the shift of hTERT transcripts to a β- pattern. Zhdanov et al. [136] used SSOs block the active sites for SRp20 or SRp40 regulatory splicing proteins. Oligonucleotide AON-Ex726 anchored to a cis-site for SRFS2 splicing regulatory proteins reduces the level of full-length hTERT and induces the β- variant in various brain cancer cell lines [137] associated with decreased proliferation and increased apoptosis levels (2 in Fig. 2).

### Phytochemicals and substances with off-target effects

Telomerase activity in many cancer types can be inhibited by a wide variety of natural herbal chemical compounds known as phytochemicals. The mode of action is only partially known and encompasses inhibition of translocation of hTERT to the nucleus; dissociation of Hsp-90 co-chaperone from hTERT; and a decrease of hTERT expression or activity [81] (Table 1).

**Table 1:** Phytochemical and other substances with off target effects on telomerase

Substance	Origin	Mode of Action	Cancer type	References
Epigallocatechin gallate	Camellia sinensis	1,2	BC, ML	[86]
Papaverine	Papaveraceae	1,2	BC	[265]
Quercetin	Tea, onion,wine, flavonoids fruits, vegetables	2,5	CA	[266]
Genistein	Secondary plant metabolites and soybean	1,2	BC	[146]
Genistein	Secondary plant metabolites and soybean	4	BC, CC, LC, OC	[267]
Curcumin	Curcuma longa	1,2	BC, leukemia	[141]
Ginsenoside Rk1	Sun Ginseng	1,2,5	LC	[268]
Allicin and ajoene	Garlic	2,5	GC	[138]
Resveratrol	Hellebores, grapes, peanuts, legumes*,* red wine	1,2		[266]
Silibinin	*Silybum marianum*	1,2	PC	[269]
Epigallocatechin gallate	Camellia sinensis	3	BT	[270]
Wortmanin	*-Pinicillium funiculosum,* *-Talaronyces wortmannii*	2	Leukemia	[271]
Rapamycin	*Streptomyces hygroscopicus*	1,2	Glioma, CC	[271, 272]
Trichostatin A	*Streptomyces platensis*	2	BT (Pediatric)	[271, 272]
1,25-dihydroxyvitamin D3	Endogenous (skin) UV exposure	1,2	OC	[273]
All-trans retinoic acid	Vitamin A derivatives	1,2	BC	[274]
Vitamin E	Plant oils	2	OC	[275]
5-Azacytidine	Synthetic chemical (analog of cytidine)	1	LC, MDS	[276]
Suramin	Synthetic chemical (chemosensitizer)	1	HECC	156]
Rapamycin (Sirolimus)	*Streptomyces hygroscopicus*	1,2	CC	[272]
Aspirin	NSAID	1,2	CC	[157]
Indomethacin	NSAID	1,2	CC	[157
Tamoxifen	Nonsteroidal antiestrogen	1	LC,BC, EMC	[277]
Melatonin	Tryptophan metabolite (hormone)	1,2	BC	[165]
Cisplatin	Chemotherapeutic drug	1,2	TC, OC, Mult	[164]
Doxorubicin	Cytotoxic anthracycline antibiotic	2	BC, Panc.C, Mult	[278]

1, Direct inhibition of hTERT activity; 2, Inhibition of hTERT synthesis; 3, Competitive binding with respect to the RNA substrate primer; 4, Disturbance in hTERT translocation to the nucleus.5, Apoptosis induction

*BC* breast cancer, *PC* prostate cancer, *ML* pro-myelocytic leukemia, *CA* colon adenocarcinoma, *CC* colorectal cancer, *LC* lung cancer, *OC* ovarian cancer, *LC* liver cancer, *BT* brain tumor, MDS, myelodysplastic syndrome, *HECC* human epithelial cell carcinoma, *EMC* endometrial cancer, *TC* testicular cancer, Mult, multiple, *Panc. C,* pancreatic cancer, *NSAID* non-steroidal anti-inflammatory drug

The substances include allicin, an organophosphate derived from garlic [138]; curcumin, a phenol present in turmeric [137–145]; the flavonolignan silbinin; an organosulfur derived from *Silybum marianum* and cruciferous vegetables; epigallocatechin gallate (EGCG), a catechin in green tea [86]. Curcumin [141], genistein [146] EGCG [147], and sulforaphane [148], were tested in breast cancer cells and the non-malignant breast cell line. The mode of action encompasses inhibition of translocation of hTERT to the nucleus [139]; dissociation of Hsp-90 co-chaperone from hTERT [143]; and a decrease of hTERT expression or activity [139–142, 149].

Numerous other drugs with off-target effects on telomerase activity (not always phytochemicals) have been identified. These include substances acting via downregulation of hTERT gene transcription, via downregulation of hTR gene transcription, downregulation of both hTERT and hTR on transcriptional level, or acting via targeting telomere structure proteins or via unknown mechanisms (Table 1). Most available drugs and substances are unspecific with a wide variety of actions and different cellular targets that may counteract replicative immortality but may also exacerbate other cancer hallmarks such as chromosomal instability.

For example, one mechanism is downregulation of hTERT gene transcription by tyrosine kinase inhibitors dasatinib, imatinib, gefitinib, and nilotinib [149–152]. Other mechanisms are inhibition of the ubiquitin/proteasome pathway by bortezomib or cell toxicity of various origins by arsenic trioxide [153], 5-azacytidine [154], temozolomide [155], and suramin [156]. Anti-inflammatory effects are caused by aspirin [157], indomethacin [157], and celecoxib [158]. Other substances include the peroxisome proliferator-activated receptor (PPAR) activator troglitazone [159]; the histone deacetylase inhibitors romidepsin [160] and vorinostat [161]; and the mTOR pathway inhibitor rapamycin [162]. The DNA topoisomerase I inhibitor beta-lapachone [163] and the DNA crosslinker cisplatin [164] act via downregulation of hTR gene transcription. The circadian rhythm hormone melatonin downregulates both hTERT and hTR on transcriptional level [165]. Other substances inhibit telomerase activity by unknown mechanisms: perifosine [166], nimesulide [167], auranofin [168], pyrimethamine [169], azidothymidine [170], octreotide [171] and ofloxacin [172]. Quinacrine, bortezomib, etoposide, and doxorubicin directly target the telomere structure proteins TRF1, POT1, shelterin, and TNKS1 [172–176]. Various drugs proposed for skin cancer therapy, including tyrosine kinase and Wnt/β-catenin signaling inhibitors, also have inhibitory effects on telomerase [53, 176–181]. This is not unexpected because telomerase enzyme activity can be post-transcriptionally regulated by the kinases c-Abl, protein kinase C, ERK1/2, and Akt [81]. Blockade of the epidermal growth factor receptor might be effective in inhibiting telomerase activity of squamous cell carcinomas, which may result in suppression of tumor growth [182] (9 in Fig. 2).

## New therapeutic options

The combination of established and new technologies opens up new possibilities for telomerase-related cancer therapy. These novel technologies include (a) the attack of targets closely related to telomeres and telomerase, the so-called shelterin complex, a strategy named telomere uncapping or deprotection [183, 184], (b) CRISPR/Cas9-based techniques [185, 186], (c) the improvement of targeted drug delivery including the use of vector systems with mild or transient gene induction [186–189], (d) novel approaches in ALT tumors*,* (e) the development of smart synergistic combinatory therapies, including the use of vectors that specifically infect tumor cells and drive cancer-specific transgene expression,, (f) Dual hybrid telomerase inhibition (5 in Fig. 2), and (g) the development of more personalized concepts using state-of-the art blood monitoring such as liquid biopsy, TESLA [190] or TERRA [191].

### Ad a) Direct attack of the shelterin complex

It has been shown in culture experiments that the DNA damage response (DDR) machinery can be activated by telomere deprotection, which then may provoke end-to-end fusions, leading to cell death in a manner that is independent of telomerase activity and telomere length [191, 192]. Telomere deprotection is induced by altering of shelterin components with TRF1 as one possible target molecule [193]. Genetic *Trf1* deletion was shown to impair the growth of lung carcinomas and to increase mouse survival independently of telomere length. Chemical inhibition of TRF1 binding to telomeres by small molecules blocked the growth of lung carcinomas by inducing a rapid DDR without affecting survival. Similarly, chemical inhibition of TRF2 dimerization can be induced by peptides that directly bind to the TRFH dimerization domain of TRF2 [194]. Treatment of HeLa cells induced DDR activation, end-to-end fusions, and cell death [194]. Moreover, a telomere-targeted telomerase-dependent nucleoside analogue, 6-thio-2′-deoxyguanosine (6-thio-dG) is incorporated into de novo synthesized telomeres, leading to telomere dysfunction in telomerase-expressing cells. Treatment leads to rapid cell death in most of the investigated cancer cell lines in vivo xenograft models [195]. This substance is considered particularly promising because it is less toxic than other telomerase inhibitors [99]. But here too good clinical data are lacking. Although more clinical research is necessary to address the efficacy of telomere-uncapping chemical inhibition in human cancer, these strategies provide promising novel opportunities for the development of anticancer agents [193] (10 in Fig. 2).

### Ad b) CRISPR/Cas9-based technologies

The system of clustered regularly interspaced short palindromic repeats (CRISPR) and CRISPR-associated protein 9 (Cas9) can be targeted to specific DNA loci via a single guide RNA (sgRNA). Depending on the presence of a protospacer adjacent motif sequence in the target DNA sequence, Cas9 can cleave double-stranded DNA. By using a system with expression of Cas9 and specifically designed sgRNA the efficient and easy manipulation of genes is possible. For example, this technique can be used to cure cancer-causing mutations [185, 186].

Cancer genomes are characterized by genetic mutations associated with carcinogenesis, progression, metastasis, and tolerance. The CRISPR-Cas9 system can be utilized for several strategies in cancer treatment. First, it is useful as a genome editing tool for genetic screens for the identification of new targets and the improvement of current therapies. Second, it can be applied for direct cancer gene therapy, mostly in combination with pharmaceutical delivery system [196]. One possibility is to use the CRISPR-Cas9 system to cure or to attenuate the effect of cancer-causing mutations [185, 186, 196]. CRISPR-Cas9 was utilized to target the *KRAS* driver mutations in vitro and in vivo [196]. Au nanoparticles were utilized to condense CRISPR-Cas9 plasmid coding sgRNA targeting *Plk*-*1*, a master regulator gene of mitosis. LC09-PPC/Cas9-VEGFA complexes facilitated selective delivery of CRISPR-Cas9 plasmid in both orthotopic osteosarcoma, resulting in VEGFA disruption [197]. Liposome/CRISPR plasmid complex has been used to downregulate DNA methyltransferase 1 (*DNMT1*) in ovarian cancer. CRISPR-Cas9-loaded exosomes were used to inhibit the expression of PARP-1, leading to the induction of apoptosis. Moreover the controllable gene regulation system Opt/Cas-Ad has been used to regulate tumor suppressor genes and to illuminate tumor cells [188]. The CRISPR-Cas9system can also be used to support natural anti-cancer strategies. Immune cells can be edited with CRISPR-Cas9 as cancer immunotherapy [198]. For example, Cas9 protein and sgRNA were co-delivered by arginine nanoparticles into macrophages to generate SIRP-α knockout macrophages and potent T cells that are resistant to exhaustion and inhibition [199]. In all clinical trials so far, the CRISPR-Cas9 system was applied to edit the autologous T cells from patients and the edited T cells were infused back into patients [200].

A CRISPR/Cas9 based technology was developed to image and manipulate cancer cells depending on telomerase and TERT expression [200–203]. There are also approaches that directly affect telomeric shortening. In a recent study, a CRISPR-Cas9 system has been created to target and remove telomere repeats [202]. In cell culture experiments, CRISRP-Cas9-mediated telomere removal led to mitochondrial dysfunction, cell growth arrest, and toxicity. For analysis, sgRNA sequence targeting telomere repeats were cloned into a lentiCRISPR-v2 construct that expresses both Cas9 endonuclease and sgRNA. This lentiCRISPR-sgRNA-telomere was introduced into cells by transient transfection. Expression of Cas9 and sgRNA-telomere led to an approximate 50% reduction of telomere amounts when compared to mock DNA transfection. There was over 70% decline in TRF band intensity but no shortening of TRF length, indicating removal of complete and intact telomeres [202]. Although, to date, there are no studies describing attempts to modulate hTERT alternative splicing with gene-editing protocols, such an approach alongside different delivery modalities may become a powerful instrument for anti-telomerase therapy.

A number of potential feasibility and safety hurdles still exist that may affect clinical applications. These include the extent of “off-target” mutations, the immunogenicity of nucleases, and delivery problems [202, 204, 205]. Notably, high-fidelity CRISPR-Cas9 nuclease variants with no or very few detectable off-target effects have recently been developed [205–208].

### Ad c) Improvement of targeted drug delivery

Possible methods for delivery of Cas9-sgRNA complex into mammalian cells are a) microinjection-based delivery, b) viral vector (AAV) based delivery, c) lipofection, or d) cell-penetrating peptides-based delivery [209]. There are challenges in delivering all the components required for editing into target cells. Gene correction by more precise Homology Directed Repair (HDR) requires donor DNA, which is more difficult to deliver than nonintegrating viral vectors or RNA transfection for genome mutation by non-homologous end joining (NHEJ). HDR appears to be less efficient in certain cell types [210, 211], although progress is being made during the last years [212]. For viral vector transfer, all systems have advantages and disadvantages. Retroviruses and lentiviruses have long-term gene expression in most cells and some oncogenic potential as a disadvantage. Adeno virus-associated viruses (AAVs) are non-inflammatory and non-pathogenic but have a small packaging capacity. Herpes simplex viruses have large packaging capacity, and a more transient gene expression but may induce immune responses [189]. Adenoviruses show efficient transduction rates in most cells but may also induce immune responses. For a combination with telomerase/telomere-associated therapy viruses with transient gene expression such as AAVs and herpes simplex viruses are of advantage. Vector systems are available that are non‐integrative and show poor immunogenicity and an excellent safety profile [189], allowing in principle a transient induction. For example, AAV9 has been shown to induce efficient transduction in a broad range of tissues, with high tropism for liver, heart, and skeletal muscle [213] and can even cross the blood–brain‐barrier [214, 215]. These non‐integrative vectors are capable of transient effect because they are lost after some time in proliferation (7 in Fig. 1).

### Ad d) Novel approaches in ALT tumors

The activation of ALT allows the cancer cells to bypass the effects of telomerase inhibition and to continue proliferating. Different strategies have been proposed to treat ALT + tumors [58]:

*ATR (Ataxia Telangiectasia and Rad3-related) inhibitors*: ATR is a kinase that functions in DNA damage response (DDR) pathways including the activation of replication stress checkpoint and HR [216]. ATR inhibitors may work in ALT + cells [217] even if the specificity for telomere maintenance mechanisms has been questioned in other studies [218]. Broader nonspecific effects are have also been described for osteosarcoma [219], Ewing sarcoma [220] and soft tissue sarcomas [221]. Co-treatment of ATR inhibitors with gemcitabine has proven to be broadly effective by increasing DNA damage [221]. In breast cancer, by contrast, TR inhibition seems to be more specific for ALT + cells [222].

*G-quadruplex (G4) ligands*: G4s are highly enriched in telomeres [223] and appear to be suitable for ALT + cells, as shown for glioma and osteosarcoma cells [223–226]. G4 ligands may induce cell death by mechanisms beyond inhibition of telomerase such as impeding telomere replication.

*DNA topoisomerase 2 (TOP2) inhibitors*: TOP2 is required for telomere-telomere recombination and chromatin maintenance, two ALT characteristics [227, 228], suggesting possible specificity for ALT. Indeed, TOP2 inhibition with genistein may kill ALT + cells and not the ALT − cells [229]. However, this finding was not confirmed with other inhibitors displaying better anti-proliferative effects in TEL + HeLa cells. Also doxorubicin, a known TOP2 inhibitor, was more effective against TEL + than ALT + pleomorphic pleomorphic LPS cells (SW872) [230]. Thus the specificity for telomere maintenance effects of TOP2 inhibitors remains unclear.

*Oncolytic virotherapy:* Promising approaches come from oncolytic virotherapeutical approaches. For example, osteosarcoma cells infected with an adenovirus that need an hTERT promotor for replication killed not only TEL + but also ALT + cell lines [231], suggesting that suppressed hTERT in ALT + cells can be reactivated in ALT + cells under certain circumstances and then be used for oncolytic virotherapy. A more specific treatment was recently proposed for a mutant herpes simplex virus type 1 (HSV-1) lacking ICP0, a protein that degrades the ALT repressor ATRX. This approach is based upon the observation that ALT + cells are commonly deficient for expression of ATRX protein and the role of ATRX in intrinsic resistance to viral infection. It was shown that this mutant HSV-1 was ten- to one thousand-fold more effective in infecting ATRX-deficient cells, which, for the first time, indicates a truly specific approach fpr ALT + tumor cells [232]. Oncolytic virotherapy has been proven safe in phase I clinical trials that should be followed up [231, 233, 234]. Another mutant HSV-1, talimogene laherparepvec, was used for the treatment of advanced melanoma [235]. Viral therapies rely on interacting with host receptors which always enables the development of resistance. Therefore, virotherapies with multiple receptor targets are desirable.

Many tumors that depend on ALT are difficult to treat and have a poor prognosis [236]. Overall, while telomerase inhibition holds promise as a therapeutic approach, it is important to consider the potential for ALT activation as a resistance mechanism. Future studies may help identify strategies to overcome ALT-mediated resistance and improve the efficacy of telomere-targeting therapies in cancer treatment.

### Ad e) Development of smart synergistic combinatory therapies

Combinations with CRISPR/Cas9-based technologies: Various combinations with CRISPR/Cas9-based technologies are conceivable: the combination of telomerase inhibition with Crisp/Cas9 induced abrupt telomere attrition using transiently effective vector systems and telomere deprotection measures; the combination of approaches with Crisp/Cas9 induced telomere attrition using vector systems that specifically infect tumor cells via hTR and hTERT promotors that are able to drive cancer-specific transgene expression [187]; the combination of oncolytic approaches with Crisp/Cas9 dependent cancer cell detetction strategies. The combination of novel Crisp/Cas9 based telomerase inhibition with conventional chemotherapies has several advantages that may significantly increase survival time. First, direct Crisp/Cas9-induced telomere attrition may overcome the problem that multiple cell divisions are necessary before inhibition of telomerase can lead to sufficient shortening of the telomere and result in cell death. Second, by the use of transient vector systems short-term repetitive telomerase inhibition are possible to maintain genetic stability, and a therapy with repetitive transient telomerase inhibition cycles helps to minimize negative effects on the immune system (and thus cancer fightening). Third, targets closely related to telomeres and telomerase such as shelterin complex associated proteins can help to treat tumors that induce alternative telomere lengthening. Fourth, Crisp/Cas9 dependent methods allow the identification of cells that use telomerase or ALT and allow a targetetd oncolytic therapies. Altogether there are many new options and a sophisticated combination of old and new methods may help to turn cancer, even if not curable, into a more chronic disease with a long survival time and to enhance both the safety and effectiveness of cancer gene therapy.

### Combinatory therapies in ALT positive cancer cells

ATRX loss of function is the strongest predisposing factor associated with the development of recombination mediated ALT and thus a molecular marker for ALT positivity [237]. It was recently shown that ATRX-aberrant cells are preferentially sensitive to the DNA topoisomerase 1 inhibitor irinotecan and poly ADP ribose polymerase (PARP) inhibitor Olaparib [99], and this dual therapy enhanced sensitivity and had a greater effect than each substance alone. The function of ATRX is not entirely clear It is a regulator of gene transcription and DNA damage repair and is enriched at the silenced allele of imprinted regions. In a patient-derived xenograft model, one cycle of combination therapy was sufficient to induce remission and to improve overall survival in ATRX-null mice [99]. A targeted combinatory ALT therapy may maximize therapeutic efficacy [99]. Thus ALT tumors prone to treatment-resistance, would profit from maximum efficacy and further research especially when substances such as olaparib and irinotecan are already approved for use in clinical medicine.

### Other combinatory therapies

Other combinations have also been studied and appear to have advantages to avoid resistances. For example, one L-ASNase resistance mechanism can be counteracted by using recently developed GCN2 inhibitors in ALL, AML, pancreatic cancer, and melanoma [237–240]. GCN2-ATF4 is a key pathway in amino acid metabolism upon starvation by L-ASNase in tumor cell lines [241]. Moreover the BTK inhibitor Ibrutinib synergizes with L-ASNase in ALL through a similar mechanism [242].

Some studies have investigted telomerase-related combination therapies with conventional methods. For example, the small molecule BIBR1532 can induce cell sensitivity to chemotherapy in MCF-7 and breast cancer stem cells [97]. A combination of RHPS4 with the mitotic spindle poison Taxol caused tumour remissions and further enhancement of telomere dysfunction [109]. Co-treatment of ATR inhibitors with gemcitabine has been proven to be broadly effective by increasing DNA damage [221].

### Ad f) Dual hybrid telomerase inhibition

Addressing telomerase as an anti-cancer target has several pitfalls: i) cellular senescence is induced only when telomeres have reached their critical length, thus telomerase inhibitors require appropriate time to become effective [243]; ii) induction of senescence can result in activation of oncogenes and/or silencing of tumor suppressor genes [244] and iii) the use of inhibitors is possibly hazardeous for highly proliferative cells such germ lines and stem cells [245]. These specific disadvantages inspired the development of substances that combine telomerase inhibition and other tumor suppressive effects in one hybrid molecule as a possible therapeutic approach. Molecular hybridization is a well-established tool with several successful applications so far [246].

Concomitant inhibition of carbonic anhydrase and telomerase: In a proof-of-concept study it was shown that the concomitant use of metalloenzyme carbonic anhydrase inhibitor, and various telomerase inhibitors merged within the same molecular scaffold and able to act on two validated targets in the same target cell is well suited as such a compound [247]. Tumor-associated carbonic anhydrases are selectively overexpressed in hypoxic solid tumors [248] and allow cancer cells to survive within a pH-dysregulated environment [249]. Two tested compounds efficiently suppressed telomerase activity in cell lysates and colon cancer cell lines, and resulted in telomere shortening, cell cycle arrest, replicative senescence, and apoptosis. Enzyme kinetics showed that these compounds are mixed-type inhibitors of the binding of DNA primers and deoxynucleoside triphosphate (dNTP) to the TL catalytic subunit hTERT. Both compounds were able to inhibit tumor growth for a sufficient period of time to cause critical telomere shortening [250]. In vivo studies have not yet been carried out with these substances.

L-asparaginase and telomerase inhibition: *Rhodospirillum rubrum* L‐asparaginase was shown to suppress telomerase activity in human T‐cell lymphoma cells [251]. L-Asparaginase however is a long-known anti-cancer drug for the treatment of acute lymphoblastic leukemia (ALL). It hydrolyses asparagine into aspartic acid and ammonia and thus reduces the bioavailability of asparagine to eradicate rapidly proliferating cancer cells. Thus inhibition of telomerase by RrA is characterized by a dual (anti‐asparaginase and anti‐telomerase) anti-cancer effect in one protein. Lymphoblasts lack the expression of asparagine synthetase, which makes them an ideal target, but recent reports suggest L-ASNase may also have clinical potential for the treatment of solid cancers. Moreover longer telomeres of lymphocytes make such cells more sustainable to all forms of L-asparaginase, and particularly the smaller RrA is less immunogenic than other asparaginases [252]. However, immunogenic and other severe adverse side effects limit optimal clinical use in all asparaginases. By use of novel formulations these limitations may be reduced. In addition, identification of L-ASNase resistance mechanisms have iniated the development of drug combinations to overcome chemoresistance.

Dual-hybrid telomerase inhibitors may also work by targeting both TERC and TERT simultaneously, disrupting the interaction between these components and inhibiting telomerase function more efficiently than targeting hTERT alone. By targeting multiple components of the telomerase complex, these inhibitors should have a more potent and selective inhibitory effect. Moreover there are a number of substances with at least two targets which, however, often attack similar structures or pathways. For example, some previously studied compounds known to act on DNA structures, such as quinolones and anilinoquinazoline derivatives are potential G4 ligands. The substance QQ58 had a remarkable antitumor effect in vitro despite poor telomerase inhibitory activity. Different targets are already known for many other substances including many phytochemicals. The mTOR inhibitor rapamycin inhibits both the PI3K-Akt-mTOR pathway and the telomerase activity [87, 253] and thus counteracts carcinogenesis by different modes of action. Substance 12459, a G-quadruplex-interacting agent, suppresses full-length hTERT and telomerase activity and induces inactive β- variant in the A549 lung carcinoma cell line [131]. The substance binds to G-rich sequences that can form G-quadruplexes thereby affecting alternative splicing toward β- formation. A similar approach was performed with compound CX-5461, which stabilizes the G-quadruplex [132] and shifts hTERT splicing pattern to an increase in β- and a decrease in the full-length expression, associated with telomerase inhibition and induction of apoptosis in glioblastoma cell lines. In general, G4 ligands may induce cell death by mechanisms beyond inhibition of telomerase such as impeding telomere replication. Moreover pyridostatin dissociates TRF2 from telomeres in cancer cells suggesting that telomestatin exerts its anticancer effect not only through inhibiting telomere elongation, but also by rapidly disrupting the capping function at the very ends of telomeres [254]. Pyridostatin also induces DNA damage at many other clusters of sequences with a propensity for G-quadruplex formation. As a result, the expression of these genes, including the proto-oncogene SRC, is modulated and a SRC reduction promotes growth arrest in human cancer cells by inducing DNA damage [255].

### Ad g) Development of more personalized concepts using state-of-the art blood monitoring

Novel technologies can be combined with novel sophisticated methods for the analysis of telomere lengths and length distribution in tumor cells or surrogate cells to ensure a more personalized therapy depending on the current telomere length and stability in circulating tumor cells. Recently Telomere Shortest Length Assay (TESLA) has been described, as a technique that detects telomeres from all chromosome ends from < 1 kb to 18 kb using small amounts of DNA [190]. With this assay average TL, as well as the percentage of the shortest telomeres can be monitored from peripheral blood. Compared with other TL measurement methods, TESLA provides more information about the shortest telomeres. TESLA is a very robust method that provides the possibility for personalized telomere/telomerase-related cancer therapy. TERRA monitoring may be helpful for detecting alternative telomere lengthening and identifying tumors that have converted telomere extension from telomerase activation to ALT. TERRA, the telomeric repeat-containing RNA that is transcribed from the subtelomeric region toward telomeres, is specifically upregulated in ALT cells [191]. Thus TERRA could serve as a marker for screening ALT cancers, and possibly also as a target for ALT cancers [256]. This monitoring can be ideally combined with liquid biopsy methods such as monitoring of circulating nucleic acids that have been suggested as potential biomarker candidates to optimize therapy.

The ALT phenotype is not universally observed in all cancers following telomerase inhibition and further research is needed to better understand the underlying factors that determine the response. Monitoring and mutation analysis of involved genes would be a reliable predictor of the ALT positivity in certain cancers and for prognosis together with other reliable ALT biomarkers, such as C-circle and ALT-associated PML nuclear bodies (APBs). Altogether this may lead to more efficacious treatments and better management strategies to meet the urgent needs of cancer patients.

### The TICCA strategy based upon current concepts

Telomerase is a vital and specific element in most cancer cells [257]. It is not an oncogene but expressed more vastly in cancer cells than any other tumor marker. Telomerase as a tumor target can be overcome by only one other mechanism: ALT. This fact limits the probability to develop resistance to telomerase-based treatments. Being expressed at very low levels in normal cells, together with the longer telomeres in normal stem cells, compared to malignant cells, telomerase inhibition offers some degree of specificity with low risk of toxicity in normal cells [258], and low risk in stem cells, provided that telomerase inhibition is time limited. Given that telomerase is a key regulator of senescence, apoptosis and immortality it is the ideal tumor target [81].

Several classes of telomerase inhibitors have been identified and investigated in vitro [259]. However, many clinical studies were disappointing. Thus far, only one therapeutic vaccine reached the clinic state (GV1001), and only one telomerase antagonist (imetelstat, GRN163L) reached the late study state, despite enormous efforts to generate telomerase vaccines, to identify telomerase inhibitors, and to develop promoter-driven cell killing systems. Some studies are promising but altogether current studies suggest that more than one mechanisms must be addressed simultaneously for complete eradication of cancer cells. For several reasons, the efficacy of telomerase inhibitors is limited. First, cancer cells treated under persistent telomerase inhibition may switch the mechanism of telomere elongation to ALT. Second, the cells can divide and form tumor nodes before their telomeres reach a critical length. Third, cancer cells can restore their telomerase activity (and the length of their telomeres) after elimination of an inhibitor. Fourth, a too aggressive therapy may damage stem cells and other replicating cells.

**Fig. 3:**
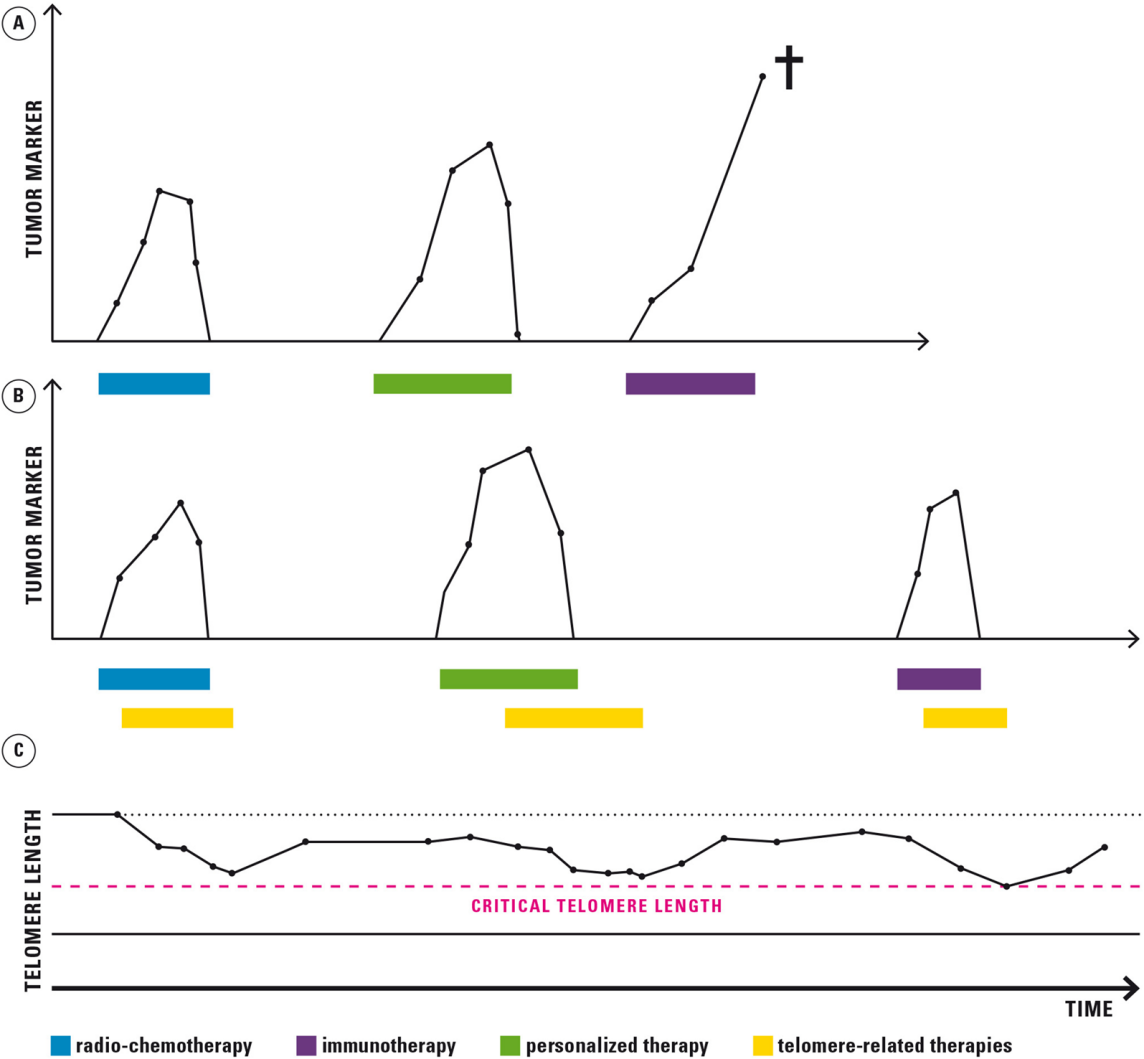
Combination of classical tumor therapies with targeted modulation of telomeres. The combination of old and new telomerase modifying technologies with conventional chemotherapies may allow (i) transient telomerase inhibition, thus keeping telomere length short, but not critically short, (ii) attack of alternative targets inducing telomere deprotection and (iii) immediate and complete telomere cleavage. Altogether these strategies may help to turn cancer, even if not curable, into a more chronic disease with a long survival time. **A** Conventional therapies alone, **B** Conventional therapies in combination with transient and targeted telomerase inhibition strategies **C**. The parallel monitoring of telomere length and structure by methods such as qPCR, TESLA or TERRA to prevent falling below critical telomere lengths and to avoid genetic instability. The lines indicate the levels of classical tumor markers such as proteins, peptides and carbohydrates found in blood, urine or tissues or novel markers such as cell-free nucleic acids and circulating tumor cells from peripheral blood (liquid biopsy). The color-coded therapies in this figure are only intended as examples and must be individually tailored to the respective tumor and patient. Different conventional therapies may be applied sequentially over a long period of time. Also, the timing of telomerase-based therapy may vary depending on the tumor and future studies.

We here summarized a number of new approaches to improve classical tumor therapies through targeted modulation of the shelterin complex. With such procedures, therapy could be personalized. Cancer, similar to chronic infectious diseases such as HIV infection, may not be cured definitively for all cases, but survival could be substantially prolonged. Based on new findings and approaches, we propose a concept how long-term survival in telomerase-based cancer therapies can be significantly improved by Transient, Immediate, Complete, and Combinatory Attack (TICCA) (Fig. 3).

### Transient attack

Highly proliferative cells such as stem cells may suffer from undesirable effects that may result from telomerase inhibition [260]. The therapy should therefore be restricted to a narrow telomere length and limited time window. On one hand, replicative senescence is tumor-suppressive [261]. On the other hand, long-term induction of telomere dysfunction and too aggressive telomere attrition may lead to chromosomal instability, which triggers chromosome fusions and activation of oncogenes and/or the silencing of tumor suppressor genes. This effect limits all strategic options and contributes to drug resistance [262].

To avoid genetic instability, a TERT-based therapy should only be used for a limited time. Smart tools have been developed for this purpose, in particular new vector systems that can be switched on and off in a targeted manner. Novel techniques have been developed for monitoring telomere length and structure to avoid over-therapy such as TESLA and TERRA [190, 191]. A time-limited use, for example as maintenance therapy after previous standard therapies is now possible. These approaches include vector systems with poor immunogenicity, improved safety profile, allowing a transient induction in a broad range of tissues, with high tropism and the possibility to cross the blood–brain‐barrier [214, 215]. Non‐integrative vectors are available that completely loose effect after some time in proliferation.

### Immediate attack

To avoid the delayed therapeutic effect of telomere shortening new methods are available for a prompt telomere related effect such as the attack of the shelterin complex by deprotection and interference with TRF1 or TRF2 [193, 194]. Nucleoside analogues are incorporated into de novo synthesized telomeres, leading to telomere dysfunction [263]. Targeted drug delivery is possible by CRISPR/Cas9-based approaches that may target the shelterin complex and/or remove telomere repeats [202, 209].

### Combinatory attack

Current caveats of telomerase inhibitors are variable response rates, the lack of specificity and the development of ALT as resistance mechanism in about 5%–10% of malignant cells.

Some studies point to the benefits of a combined attack on telomerase dependent structures. This includes the combination of telomerase inhibitors with other clinically proven therapies such as chemotherapy (with BIBR1532) [97], the combination of RHPS4 with taxol [109] or co-treatment of ATR inhibitors with gemcitabine [221]. In particular, dual-hybrid therapeutics are an example for promising novel developments e.g. the use of substances that combine telomerase inhibition and other tumor suppressive effects in one hybrid molecule such as inhibition of carbonic anhydrase or L-asparaginase [246, 249]. These substances attack two targets simultaneously in the same cell. Good progress has also been made in targeting ALT positive tumors by use of combinatory therapies that help to avoid the development of tumor resistance. The use of substances with several weaker telomere-associated targets or one main and a several weaker targets such as G4 ligands [100], quinolones, anilinoquinazoline derivatives, rapamycin, pyridostatin and telomestatin may be used for maintenance therapies [264].

### Complete attack

The goal of any tumor therapy is to kill as many tumor cells as possible. All tumor cells either directly depend on telomerase activity or telomerase can even be reactivated in ALT positive cells, as studies have shown [231], indicating that the complete removal of all tumor cells can theoretically be achieved with only one target: telomerase.

An armada of methodologies is available including oligonucleotide inhibitors, small molecule hTERT inhibitors, G4-stabilizing compounds with improved selectivity [223–226], immunotherapeutic approaches [118–122], telomerase-directed gene therapy using telomerase promoters of cancer cells as targets [130], conventional and Crisp/Cas9 induced alternative splicing [88, 89, 132], alternative splicing with splice-switching oligonucleotides [133, 134–137, 206, 208], the use of phytochemicals with a wide variety of different targets [257]; Crisp/Cas9 based and other telomere deprotection measures [183, 184, 187]; the use of ATR inhibitors [217, 218–221]. DNA topoisomerase 2 (TOP2) inhibitors [229, 230] and poly ADP ribose polymerase (PARP) inhibitors (that may work in ALT + cells) [99] and targeted oncolytic virotherapy with ALT specific targets such as HSV-1 lacking ICP0 [232].

### Personalized approach

In order to use these methods in a targeted manner the development of more personalized concepts using classical tumor markers such as CA-125 and state-of-the art monitoring such as liquid biopsy, and analyses of mutations and the tumor histology, under supervision of specialized tumor board experts from different sub-specialities, is required. Various tools are available for detecting telomere length and ALT status and mutational analysis including Telomere Shortest Length Assay (TESLA) as a technique that detects telomeres from all chromosome ends from < 1 kb to 18 kb using small amounts of DNA [190]; TERRA (the telomeric repeat-containing RNA) monitoring for the detection of ALT cancers [191], other reliable ALT biomarkers, such as C-circle and APBs [58] and next generation sequencing methods. TERRA could serve as a marker for screening ALT cancers, and possibly also as a target for ALT cancers. This monitoring can be ideally combined with liquid biopsy methods such as monitoring of circulating nucleic acids.

Personalized concepts also include CRISPR/Cas9-based technologies for individual gene correction by more precise Homology Directed Repair (HDR) [209–212], the identification of tumor cells and individual editing of autologous T cells to generate potent T cells that are resistant to exhaustion [199, 200]. The use of individual therapies in individual resistance states such as GCN2 or BTK inhibitors in L-ASNase resistant patients should be taken into consideration [237–240, 242]. A consequent monitoring and telomere-dependent adaptation of procedures may substantially prolong survival. We recommend a protracted approach, as described in Fig. 3, whereby studies must show which setting is most suitable for which tumor to be adapted accordingly.

In conclusion, a wide variety of new methods is available including the use of the CRISPR-Cas9 systems to cure or to attenuate the effect of cancer-causing mutations or to directly affect telomeric shortening, altenative splicing, dual hybrid inhibitors and G quadruplex stabilzers. The combination of these methods with conventional chemotherapies can overcome the problem that multiple cell divisions are necessary before inhibition of telomerase leads to sufficient shortening of the telomere. A therapy with repetitive transient telomerase inhibition cycles using transient vector systems minimizes negative effects on the immune system and helps to maintain genetic stability. Targets closely related to telomeres and telomerase such as shelterin complex proteins and selective targeting for ALT tumors helps to avoid tumor resistances. A supportive effect of telomerase inhibition in combination with any conventional chemotherapy is likely effective and it is for the first time conceivable that survival time can be significantly prolonged by transient telomerase inhibition.

## Data Availability

All data are shown in the manuscript.

## Abbreviations

AAV: Adeno-Associated Virus
Akt: Protein kinase B
ALT: Alternative lengthening of telomeres
CRISPR/Cas9: Clustered regularly interspaced short palindromic repeats and CRISPR-associated protein 9
CTC1: Telomere protection component 1
DDR: DNA damage response
DNMT1: DNA (cytosine-5)-methyltransferase 1
EGCG: Epigallocatechin-3-gallate
HIV: Human immunodeficiency virus
hTERT: Human Telomerase Reverse Transcriptase
hTR: Human telomerase RNA
KRAS: Kirsten rat sarcoma virus
LC09: Osteosarcoma cell-specific aptamer
MHC: Major histocompatibility complex
mTOR: Mammalian Target of Rapamycin
PI3K: Phosphoinositide-3-kinase
Plk1: Polo-like-Kinase 1
POT1: Protection of telomeres protein 1
PPAR: Peroxisome proliferator-activated receptor
PPC: PEG (polyethylene glycol)-PEI (poly-ethylene imine) Cholesterol
RAP1: Repressor/activator protein 1
sgRNA: Single guide RNA
STN1: Suppressor of cdc thirteen 1
TEN1: Telomeric pathway with STN1
TERC/TR: Telomerase RNA component
TERRA: TElomeric Repeat-containing RNA
TESLA: Telomere Shortest Length Assay
TIN2: TRF1- and TRF2-Interacting Nuclear Protein 2
TL: Telomere length
TPP1: TINT1/PTOP/PIP1 protein
TRF: Telomeric-repeat binding factor (TRF1, TRF2)
TRFH: TRF homology domain
VEGFA: Vascular endothelial growth factor-A
6-thio-dG: 6-Thio-2´-deoxyguanosine
